# Curcumin Induced Human Gastric Cancer BGC-823 Cells Apoptosis by ROS-Mediated ASK1-MKK4-JNK Stress Signaling Pathway

**DOI:** 10.3390/ijms150915754

**Published:** 2014-09-05

**Authors:** Tao Liang, Xiaojian Zhang, Wenhua Xue, Songfeng Zhao, Xiang Zhang, Jianying Pei

**Affiliations:** Department of Pharmacy, the First Affiliated Hospital of Zhengzhou University, Zhengzhou 450052, China; E-Mails: liangtaozhz@163.com (T.L.); xuewenhua77@163.com (W.X.); zhaosf450@126.com (S.Z.); zhangxiang028@126.com (X.Z.); jianyingpei@126.com (J.P.)

**Keywords:** curcumin, gastric cancer, ROS, ASK1-MKK4-JNK, apoptosis

## Abstract

The signaling mediated by stress-activated MAP kinases (MAPK), c-Jun *N*-terminal kinase (JNK) has well-established importance in cancer. In the present report, we investigated the effects of curcumin on the signaling pathway in human gastric cancer BGC-823 cells. Curcumin induced reactive oxygen species (ROS) production and BGC-823 cells apoptosis. Inhibition of ROS generation by antioxidant (NAC or Trion) significantly prevented curcumin-mediated apoptosis. Notably, we observed that curcumin activated ASK1, a MAPKKK that is oxidative stress sensitive and responsible to phosphorylation of JNK via triggering cascades, up-regulated an upstream effector of the JNK, MKK4, and phosphorylated JNK protein expression in BGC-823 cells. However, curcumin induced ASK1-MKK4-JNK signaling was attenuated by NAC. All the findings confirm the possibility that oxidative stress-activated ASK1-MKK4-JNK signaling cascade promotes the apoptotic response in curcumin-treated BGC-823 cells.

## 1. Introduction

**Figure 1:**
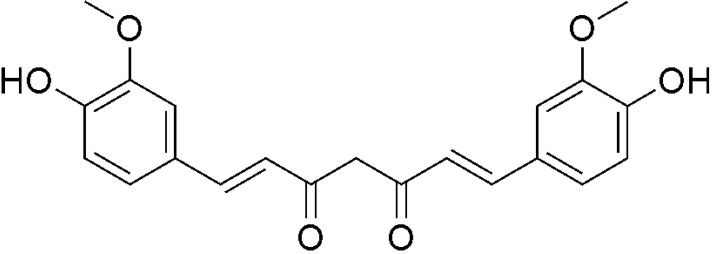
Chemical structure of curcumin.

Gastric cancer is one of the most common malignant cancers with unfavorable prognoses and high mortality rates worldwide [1]. Although recent breakthroughs in therapy and diagnosis, treatment progress of gastric cancer remains limited. The survival rate is low, and most diagnosed patients are incurable [2]. Chemoprevention is an important way in gastric cancer prevention before it occurs [3]. Natural products and their derivatives, such as green tea, resveratrol, and vitamins, have potential benefits due to their chemoprevention [4]. Curcumin (Figure 1), a natural biologically chemopreventive agent, extracted from rhizomes of curcuma species, possesses antitumor, antioxidant and antiproliferative activities [5]. Additionally, curcumin showed potential bioavailability in inhibiting gastric cancer cell proliferation [6,7,8]. How curcumin mediates its anticancer effect is not completely understood. However, growing reports have shown that reactive oxygen species (ROS) generation plays a critical role in determining the curcumin-induced cancer cells apoptosis [9,10,11].

ROS functions as byproducts of the normal cellular metabolism of oxygen [12], however, a dramatic increase in ROS levels can cause oxidative stress. When cells expose to stress stimuli, the MAPK cascade (MKKK/MKK/MAPK) is rapidly activated. Each MAP kinase kinases (MKK) is regulated by multiple MAP kinase kinase kinase (MKKK) proteins. Sequentially, MKK proteins phosphorylate the downstream MAPK. Eventually, the activation of MAPK signaling proteins were involved in apoptosis via stress stimuli [13,14,15]. The c-Jun *N*-terminal Kinase (JNK), a member of the mitogen-activated protein kinases (MAPKs), is required for stress-induced apoptosis [16,17]. The present study confirmed that curcumin inhibited cell proliferation and caused BGC-823 cell apoptosis via mediating ROS-mediated ASK1-MKK4-JNK signaling pathways.

## 2. Results and Discussion

### 2.1. Curcumin Inhibited Cell Proliferation in BGC-823 Cells

**Figure 2:**
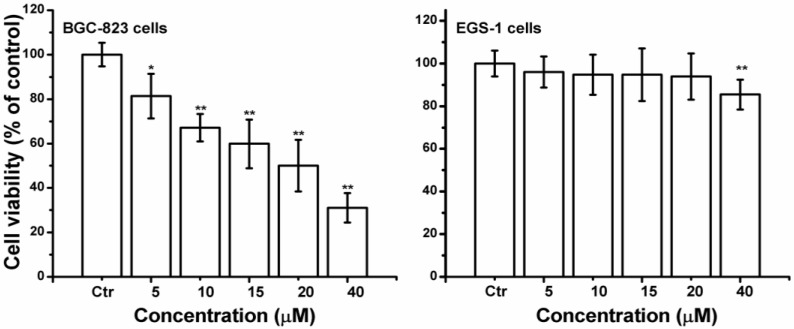
Effects of curcumin on cell growth of BGC-823 or EGS-1 cells. The MTT staining assay was used to assess cell viability. After 24 h treatment, curcumin (0–40 µM) significantly decreased of BGC-823 or EGS-1 cells cell viability with a concentration-dependent manner. The value was expressed as means ± S.D. of three independent experiments. * *p* < 0.05, ** *p* < 0.01 compared with the control (0 µM) group.

Curcumin significant inhibited cell growth in a concentration-dependent manner, and the cell viability of 20 or 40 µM curcumin treated BGC-823 cells was decreased by 50.0% or 68.94% respectively (Figure 2). While curcumin (0–20 µM) did not significantly affect the human gastric epithelial cells EGS-1. Owing to the prominent proliferation inhibition of BGC-823 cells, 20 µM of curcumin was used for most of the subsequent assays, and the concentration was close to the IC_50_ value of BGC-823 cells.

### 2.2. Curcumin Induced Oxidative Stress in BGC-823 Cells

**Figure 3:**
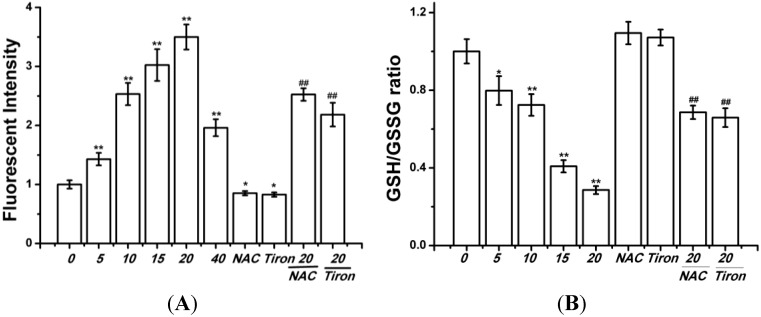
Effects of curcumin on redox state in BGC-823 cells. (**A**) The H_2_DCFDA probe was used to determine ROS level; (**B**) The GSH/GSSG ratio was used to measured oxidative stress. Control group (0 µM) level was represented as “1.0”. The value was expressed as means ± S.D. of three independent experiments. * *p* < 0.05, ** *p* < 0.01 compared with the control (0 µM) group; ^##^ *p* < 0.05 compared with the curcumin-treated (20 µM) group.

Previous experiments have demonstrated that curcumin could induce earlier ROS production, we also investigated the change of ROS level in curcumin-treated BGC-823 cells. As shown in Figure 3A, curcumin (0, 5, 10, 15, or 20 µM) enhanced the levels of ROS in BGC-823 cells. In addition, the BGC-823 cells were exposed to curcumin for 24 h (0, 5, 10, 15, or 20 µM), the GSH/GSSG ratio decrease (Figure 3B). The ROS scavenger *N*-acetylcysteine (NAC), and Tiron (a vitamin E analog) effectively attenuated the ROS production in curcumin (20 µM) treated BGC-823 cells, and the GSH/GSSG ratio was reduced (Figure 3).

### 2.3. Curcumin-Triggered Apoptosis in BGC-823 Cells Was Related with the ROS Production in BGC-823 Cells

**Figure 4:**
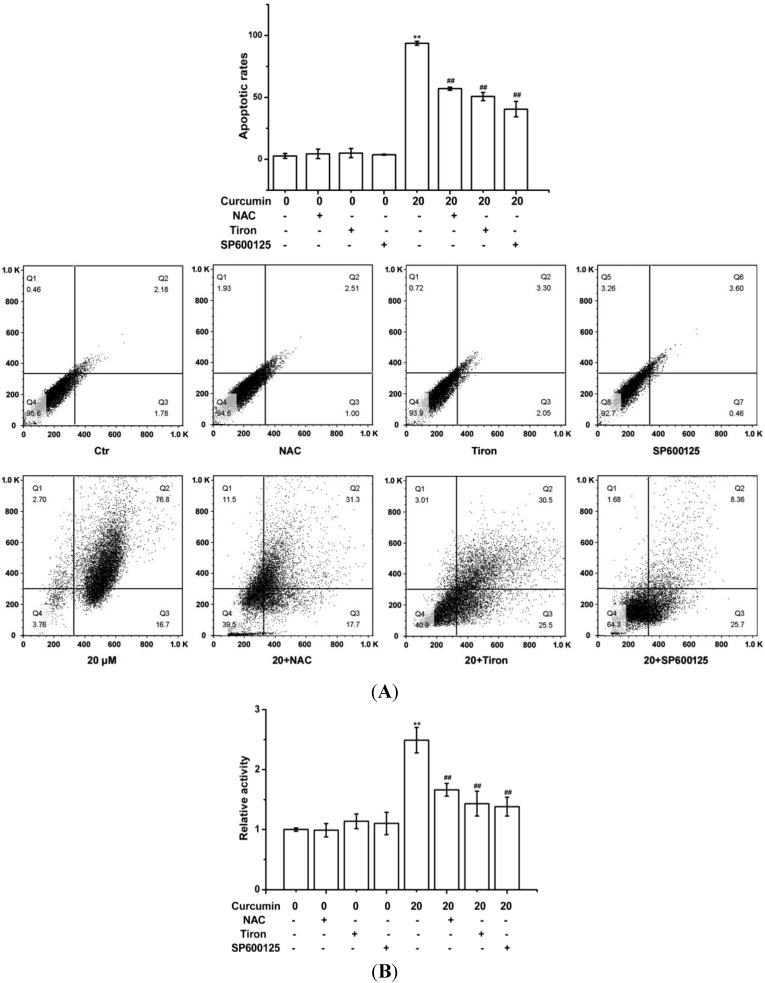
Curcumin triggered apoptosis in BGC-823 cells. The indicated amounts of curcumin treated BGC-823 for 24 h with or without NAC (400 µM), Tiron (400 μM) or JNK inhibitor (SP600125, 20 μM). (**A**) Apoptotic rates were analyzed quantitatively via flow cytometry; (**B**) Caspase-3 activity was examined using colorimetric kit assay, with the relative intensity representing the result of caspase-3 activity. The value was expressed as means ± S.D. of three independent experiments. ** *p* < 0.01 compared with the control (0 µM) group; ^##^ *p* < 0.01 compared with the curcumin-treated (20 µM) group.

The increase of apoptotic rates and the activation of caspase-3 provided supports for the apoptosis induced by curcumin (Figure 4). And the association of apoptosis to ROS generation by curcumin was further confirmed by pre-treating BGC-823 cells with antioxidant NAC or Tiron. The results showed ROS scavengers (NAC or Tiron) effectively reduced curcumin-induced apoptotic responses (Figure 4). In addition, JNK inhibitor (SP600125, 20 μM) attenuated the increase of apoptotic rates and the activation of caspase-3 (Figure 4).

### 2.4. Curcumin Activated ROS-Mediated JNK Cascade in BGC-823 Cells

**Figure 5:**
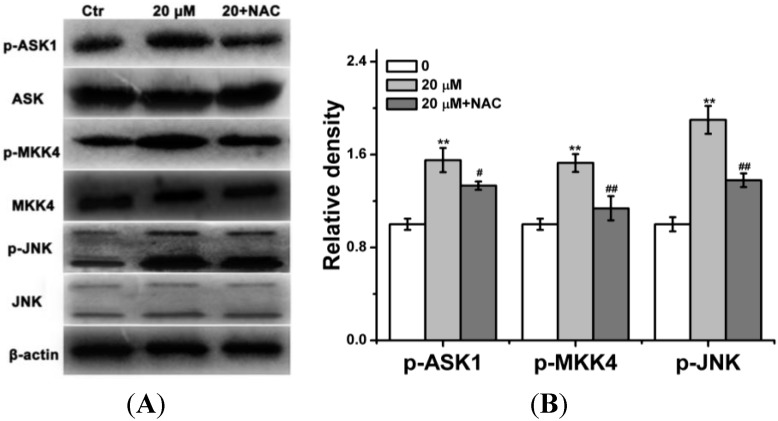
Curcumin activated JNK signaling pathway in BGC-823 cells. The amounts of curcumin (0 or 20 µM) treated BGC-823 for 24 h with or without NAC (400 µM). Then Western blot was used to assess the expression of antibodies. (**A**) p-ASK1, p-MKK4, p-JNK, total ASK1, total MKK4 and total JNK were analyzed via Western blot; (**B**) The expressions of p-ASK1, p-MKK4, p-JNK, total ASK1, total MKK4 and total JNK protein were quantitative. Control group (0 µM) level was represented as “1.0”. The value was expressed as means ± S.D. of three independent experiments. ** *p* < 0.01 compared with the control (0 µM) group; ^#^ *p* < 0.05, ^##^ *p* < 0.05 curcumin-treated (20 µM) group.

As JNK inhibitor prevented *curcumin*-induced apoptosis and caspase-3 activation were observed in our study, and the JNK cascade is pivotal for apoptosis induced by stress stimuli, we next to investigate whether curcumin could activate of JNK cascade including MAP3K (ASK-1), MAP2K (MKK4), and MAPK (JNK) in BGC-823 cells. As shown in Figure 5, curcumin (20 μM) upregulated the phosphorylation of ASK-1, MKK4 and JNK in BGC-823 cells. In order to explore the relationship between ROS and JNK cascade in curcumin-treated BGC-823 cells apoptosis, the ROS scavenger NAC was used. The results indicated that the pre-treatment with NAC prevented the phosphorylation of ASK-1, MKK4 and JNK in curcumin-treated BGC-823 cells (Figure 5).

### 2.5. Discussion

Curcumin, a natural anticancer agent, has been widely noted due to its potent inhibitory action on tumorigenesis and its activity of cancer chemoprevention by inducing apoptosis [18,19,20]. As it is widely taken orally as an edible agent, curcumin also has been considered as an option for preventing and treating gastric cancer. For instance, curcumin could suppress human gastric cancer cells proliferation via series of biological pathways including mutagenicity [21], cell cycle regulation [22,23], apoptosis [24,25], tumorigenesis [26], angiogenesis [27], and invasion [28]. Curcumin also exhibited potent chemosensitization through downregulating the NF-κB in human gastric carcinoma cells SGC-7901 and AGS [29,30]. Even though many of mechanisms of the compound have been researched, much remains to be investigated for its role of gastric cancer. The most important findings of this study was that curcumin activated ROS-mediated ASK1-MKK4-JNK signaling pathway, ultimately induced human gastric cancer BGC-823 cells apoptosis.

ROS are oxygen-derived free radicals, that are endogenously produced by mitochondria, NAD(P)H oxidase systems or enzymes, such as xanthine oxidases, cytochrome p450, and cyclooxygenases [31]. However, oxidative stress, caused by ROS overproduction, will leed to cellular damage, including DNA lesions, protein oxidation, and lipid peroxidation, it can also promote tumorigenesis [32]. It is well known that natural compounds can reverse, suppress, or prevent the development of cancer, in spite of the fact that exact mechanisms of action are not clearly explained [33]. Induction of apoptosis seems to be a potential anti-cancer approach of flavonoids in numerous cellular systems, more importantly, many of these compounds act as an important prooxidants inducing different tumor cells apoptosis [34,35]. Previous studies demonstrated that ROS production played a vital role in curcumin-triggered apoptosis in some cells [9,10,11], and ROS-related apoptosis was also observed in curcumin-treated BGC-823 cells, evidenced by the production of intracellular ROS (Figure 3A), and the decrease of GSH/GSSG ratio (Figure 3B), and pre-treatment with NAC or Tiron reversed the apoptosis (Figure 4), indicating that curcumin can induce common cellular physiological toxic responses in different cancer cell lines. Of note, after curcumin (40 µM) treatment, the cell viability (Figure 2) and the ROS level (Figure 3A) were decreased in BGC-823 cells, as well as the cell viability of human gastric epithelial cells EGS-1 (Figure 2), implying curcumin’s (40 µM) limited efficacy in ROS production maybe becomes its toxicity.

The proteins of mitogen-activated protein kinase (MAPK) family are conserved signal transduction pathways that are activated by extracellular stimuli [36,37]. In particular, c-Jun *N*-terminal kinase (JNK), also termed stress-activated protein kinase (SAPK) pathway, is inducted by stress-related stimuli, which correlated with activation of apoptosis [16,17]. In this regard, results showed that pre-treating BGC-823 cells with SP600125 (JNK inhibitor) marked attenuated the enhancement of apoptotic rates and the activation of caspase-3, suggesting JNK might be involved in curcumin-induced apoptotic responses (Figure 4). In accordance with our results, previous studies demonstrated curcumin treatment induced apoptosis by activating JNK in tumor cells [38,39]. Reports also certified curcumin induced p38-MAPK activation in human ovarian cancer cells [40] and in human hepatocellular carcinoma Huh7 cells [41]. Accordingly, ERK activation by curcumin was observed in human leukemia THP-1 cells [42].

Since the sequential phosphorylation of downstream proteins activate MAPK signaling pathway, the possible contribution of JNK cascade was studied next. The findings showed that ASK1-MKK4-JNK cascade was activated in curcumin-treated BGC-823 cells (Figure 5). Because both JNK activation and ROS increase were found in curcumin-induced BGC-823 cells apoptosis, we sought a mechanism that may explain this phenomenon. Here, the results showed that curcumin phosphorylated of ASK-1, MKK4 and JNK, but the activation of ASK1-MKK4-JNK cascade was effectively inhibited by NAC, indicating that the production of ROS in BGC-823 cells resulted in the signaling pathway activation (Figure 5).

## 3. Experimental Section

### 3.1. Materials

Curcumin, dimethylsulfoxide (DMSO), *N*-acetylcysteine (NAC), vitamin E analog Tiron, Annexin V/FITC apoptosis detection kit, and 2',7'-dichlorodihydrofluorescein diacetate (H_2_DCFDA) molecular probes were purchased from Sigma-Aldrich Co. LLC. (St. Louis, MO, USA). Dulbecco’s modified Eagle’s medium (DMEM) and Fetal bovine serum (FBS) were purchased from Grand Island Biological Company (Grand Island, NY, USA). JNK inhibitor (SP600125) was purchased from Santa Cruz Biotechnology Inc. (Santa Cruz, CA, USA). The purity of curcumin powder (Sigma-Aldrich, St. Louis, MO, USA) was ≥98%.

### 3.2. Effect of Curcumin on Cell Viability

Human gastric cancer cell line BGC-823 and the human gastric epithelial cell line GES-1 were purchased from Cell Bank of the Committee on Type Culture Collection of Chinese Academy of Sciences (Shanghai, China). The BGC-823 or GES-1 cells grown seeded (6 × 10^3^ cells/well) in DMEM with 10% FBS at 37 °C with 5% CO_2_. The effect of curcumin on cell viability was investigated via the 3-(4,5-dimethylthiazol-2-yl)-2,5-diphenyl-tetrazolium bromide (MTT) assay [43,44]. Briefly, cells were cultured with or without indicated amounts of curcumin (0, 5, 10, 15, 20 or 40 µM) for 24 h. Medium was removed, and 20 µL of MTT (5 mg/mL) was added. After 4 h, the medium was aspirated, and 150 µL dimethyl sulfoxide (DMSO) was added. A fluorescent plate reader (Millipore Corp., Bedford, MA, USA) was used to measure absorbance intensity at 570 nm. The values were represented as percent cell viability relative to the control group.

### 3.3. Detection of ROS Level

Fluorogenic probe 2',7'-dichlorodihydrofluorescein diacetate (H_2_DCFDA) were used to assess the production of ROS [45]. In Brief, BGC-823 cells were incubated with the increasing concentrations of curcumin with or without NAC (400 µM) or Tiron (400 µM) for 1 h. Then 30 μM of H_2_DCFDA was added. After 30 min, stained cells were washed, and detected using the fluorescent plate reader (λEX/λEM = 485 nm/535 nm). ROS production was represented as the percentage relative to untreated control cells.

### 3.4. Measurement of GSH/GSSG Ratio

GSH/GSSG ratio was investigated to assess the oxidative stress. T-GSH was measured with DTNB (5,5-dithio-bis(2-nitrobenzoic)) according to the method described previously [45,46]. The cells were treated with 5% 5-sulfosalicylic acid for 30 s, the centrifuged for 10 min. The resultant extract was assayed, and the reduced GSH concentration was obtained by quantifying the reduction of DTNB through its conversion to 5-thio-2-nitrobenzoic acid (TNB) at 412 nm.

### 3.5. Apoptotic Effect of Curcumin Is Assayed by Flow Cytometry

Flow cytometry was used to determine whether the curcumin could induce BGC-823 cells apoptosis. After curcumin with or without NAC (400 µM) or Tiron (400 µM) treatment for 24 h, the cells were harvested and washed twice with ice-cold PBS, then annexin V-FITC (5 μL) and PI (1 mg/mL, 1 μL) were then added to the cells. The samples were analyzed using a flow cytometer (BD, Franklin Lakes, NJ, USA).

### 3.6. Effect of Curcumin on Caspase-3 Activity

Caspase-3 activity was measured through a colorimetric assay according to the manufacturer’s instructions (BioVision, Milpitas, CA, USA). The kit utilizes synthetic tetrapeptides labeled with pnitroaniline (pNA). Briefly, cells were lysed, and the supernatants were harvested and fostered with the reaction buffer, which contained dithiothreitol and the caspase-3 substrate Asp-Glu-Val-Asp (DEVD)-*p*-nitroaniline (pNA) at 37 °C. Absorbance at 405 nm was measured spectrophotometrically by the ELISA reader [47].

### 3.7. Western Blot Analysis

Cold RIPA buffer (50 mM Tris-HCl, pH 7.5, 1% NP-40, 0.5% sodium deoxycholic acid, and 0.1% SDS) containing proteinase inhibitors (1 mM phenylmethylsulfonyl fluoride, 2 μg/mL aprotinin, and 2 μg/mL leupeptin) was used to lyse cells, then the cells was centrifuged at 4 °C at 14,000× *g* for 20 min [48]. Extracted with the total cellular protein and assayed the protein concentration using a SmartSpec Plus Spectrophotometer (Bio-Rad Lab., Hercules, CA, USA). Equal amounts of protein (40 µg) was separated by sodium dodecyl sulfate-polyacrylamide gel electrophoresis (SDS-PAGE), transferred onto nitrocellulose membranes (Schleicher and Schuell, Keene, NH, USA), and incubated with primary antibodies against p-ASK1 (Thr-845), p-MKK4 (Thr-261), p-JNK (Thr-183/Tyr-185), total ASK1, total MKK4 and total JNK. Enhanced chemiluminescence system was used to detect immunoreactive proteins with horseradish peroxidase (HRP)-conjugated secondary antibody. HRP-conjugated secondary antibody was measured by enhanced chemiluminescence (ECL, Thermo Scientific, Waltham, MA, USA) and were exposed using film exposure [49].

### 3.8. Statistical Analysis

Results were presented as means ± S.D. and statistical significance of differences between the treatment groups and the controls were evaluated through the analysis of variance (ANOVA) followed by student’s *t*-test, and *p* < 0.05 was considered statistically significant. The analyses were performed using the SPSS 17 software (SPSS Inc., Chicago, IL, USA, 2008).

## 4. Conclusions

In conclusion, the results demonstrated that curcumin could induce ROS-mediated ASK1-MKK4-JNK cascade, and lead to BGC-823 cell apoptosis. In addition, these results provided potential therapeutic effect of curcumin against human gastric cancer. However, further studies about the anticancer mechanisms need to be addressed.
